# Non-invasive assessment of periodontal inflammation by continuum-removal hemodynamic spectral indices

**DOI:** 10.1186/s40001-024-01748-0

**Published:** 2024-03-25

**Authors:** Yuan Guo, Yixiang Huang, Changping Huang, Xuejian Sun, Qingxian Luan, Lifu Zhang

**Affiliations:** 1 Department of Periodontology, Peking University School and Hospital of Stomatology & National Clinical Research Center for Oral Diseases & National Engineering Laboratory for Digital and Material Technology of Stomatology & Beijing Key Laboratory of Digital Stomatology, Beijing, 100081 China; 2 Second Dental Center, School and Hospital of Stomatology, Peking University, Beijing, 100081 China; 3 Aerospace Information Research Institute, Chinese Academy of Sciences, Beijing, 100094 China; 4 University of Chinese Academy of Sciences, Beijing, 100049 China

**Keywords:** Periodontitis, Optical spectroscopy, Inflammation, Tissue oxygenation, Continuum-removal spectra

## Abstract

**Background:**

Hyperspectral techniques have aroused great interest in non-invasively measuring periodontal tissue hemodynamics. However, current studies mainly focused on three typical inflammation stages (healthy, gingivitis and periodontitis) and practical approaches for using optical spectroscopy for early and precisely detection of periodontal inflammation at finer disease stages have not been well studied.

**Methods:**

This study provided novel spectroscopic insights into periodontitis at different stages of disease, and developed six simple but physically meaning hemodynamic spectral indices (HSIs) including four spectral absorption depths of oxyhemoglobin ($D_{{{\text{HbO}}_{2} }}$), deoxyhemoglobin ($D_{{{\text{Hb}}}}$), total hemoglobin ($t{\text{Hb}}$) and tissue water ($D_{{{\text{water}}}}$), and two normalized difference indices of oxyhemoglobin($ND{\text{HbO}}_{2} I$) and deoxyhemoglobin ($ND{\text{Hb}}I$) from continuum-removal spectra (400–1700 nm) of periodontal tissue collected from 47 systemically healthy subjects over different severities from healthy, gingivitis, slight, moderate to severe periodontitis for early and precision diagnostics of periodontitis. Typical statistical analyses were conducted to explore the effectiveness of the proposed HSIs.

**Results:**

$D_{{{\text{Hb}}}}$ and $t{\text{Hb}}$ exerted significant increasing trends as inflammation progressed, whereas $D_{{{\text{HbO}}_{2} }}$ exhibited significant difference (*P* < 0.05) from the healthy sites only at moderate and severe periodontitis and $D_{{{\text{water}}}}$ presented unstable sensitives to disease severity. By contrast, $ND{\text{HbO}}_{2} I$ and $ND{\text{Hb}}I$ showed more steadily downward trends as severity increased, and demonstrated the highest correlations with clinical gold standard parameters. Particularly, the proposed normalized HSIs ($ND{\text{HbO}}_{2} I$ and $ND{\text{Hb}}I$) yielded high correlations of − 0.49 and − 0.44 with probing depth, respectively, far outperforming results achieved by previous studies. The performances of the HSIs were also confirmed using the periodontal therapy group.

**Conclusions:**

These results indicated great potentials of combination optical spectroscopy and smart devices to non-invasively probe periodontitis at earlier stages using the simple and practical HSIs.

*Trial registration* This study was retrospectively registered in the Chinese Clinical Trial Registry on October 24, 2021, and the clinical registration number is ChiCTR2100052306

## Introduction

Periodontal diseases, including gingivitis and periodontitis, are primarily inflammatory disorders of the soft and hard tissues initiated by pathogenic bacteria surrounding the teeth [1]. Gingivitis is a reversible infectious disease of gingiva, while periodontitis is an infectious, destructive inflammatory disease of periodontal tissues characterized by the formation of periodontal pockets, loss of clinical attachment and alveolar bone absorption [2]. Increasing evidence have confirmed that the oxygen content is closely related to the depth of the periodontal pocket, and the oxygen adequacy in the gingival tissue has a significant impact on the oxygen tension of the periodontal pocket [3, 4]. As the main cause of edentulism, periodontitis has been considered to be closely associated with systemic health risks, such as increased risk of cardiovascular diseases, diabetes mellitus and low birth weight/ preterm delivery [5–7]. Currently, the diagnosis of periodontitis is mainly based on clinical manifestations measured by clinical examination and X-ray film. Clinically, the bleeding index on probing (BOP), probing depth (PD) and clinical attachment loss (CAL) were widely used as gold standard to define the extent and severity of periodontitis [1]. However, this destructive method often causes strong discomfort to the patients as well as reflects cumulative effects of the host defense responding to periodontal pathogens. Given that, complementary diagnostic and prognostic methods have been sought and applied in the detection of periodontitis markers in clinical work [1, 8]. The visible and near-infrared spectroscopy (VNIR) is a promising auxiliary diagnostic and relatively low-cost technique due to its non-invasive measurement of local tissue hemodynamic changes caused by the disease [9, 10]. The rationale is that the VNIR spectra over different wavelengths ranging from 400 to 1700 nm convey unique information on regional tissue and its hemodynamics, including oxygenated hemoglobin (HbO_2_) and deoxygenated hemoglobin (Hb) in the capillary bed of tissue, and tissue edema (tissue water content), which are commonly considered as markers of tissue inflammation [11, 12]. Based on the principle, the VNIR spectroscopy has been first applied by Jobsis in 1977 to investigate cerebral and myocardial oxygen sufficiency [13]. During the past decade, the VNIR spectroscopy has been widely used in biomedical problems including muscle oxidative metabolism, exercise physiology and neuroscience and the functional activation of human cerebral cortex [14–16]. Many researches on cancer diagnostics, hemodynamic changes in the early post-burn period in burn victims, and anemia detection [17] have been conducted by measuring tissue oxygen saturation, hemoglobin volume and tissue oxygen consumption [14, 18–20].

Recently, this non-invasive method has been proposed to measure tissue hemodynamics in periodontal inflammation. As one of the pioneers, Liu et al. demonstrated the potential of using the VNIR spectroscopy to represent periodontal inflammation in vivo [9, 21]. Optical spectroscopy was also verified to determine tissue oxygenation profiles of healthy and diseased sites in smokers and to detect alterations around diseased peri-implant sites [22, 23]. However, previous studies mainly focused on roughly three typical stages of inflammation development (i.e., healthy, gingivitis and periodontitis) and practical approaches for using optical spectroscopy for early and precisely detection of periodontal inflammation at finer disease stages have not been well studied. To this end, we provided increased insights into spectral fingerprints over periodontal sites at different stages of disease development from healthy, gingivitis to slight, moderate and severe periodontitis. On this basis, six hemodynamic spectral indices (HSIs) closely related to inflammation hemodynamics, including four spectral absorption depths of oxyhemoglobin ($D_{{{\text{HbO}}_{2} }}$), deoxyhemoglobin ($D_{{{\text{Hb}}}}$), total hemoglobin ($t{\text{Hb}}$) and tissue water ($D_{{{\text{water}}}}$), and two normalized difference indices of oxyhemoglobin($NDHbO_{2} I$) and deoxyhemoglobin ($NDHbI$), were developed from continuum removal based reflectance spectra to build linkage with periodontal clinical gold standard parameters. The effectiveness and simplicity of these proposed non-destructive HSIs were thoroughly evaluated and may offer great potentials of combination optical spectroscopy and smart devices to non-invasively probe periodontal diseases at earlier stages by simple but physically meaning hemodynamic spectral indices derived from continuum removed reflectance spectroscopy.

## Methods

### Study subjects

The research protocol was documentarily approved by the Ethics Committee of Peking University Stomatology Hospital (PKUSSIRB-202167120) and has been registered in the Chinese Clinical Trial Registry (ChiCTR2100052306). All procedures performed in this study involving human participants were in accordance with the Helsinki Declaration of 1975. An observational study conforming to STROBE guidelines was conducted. The informed, written consent was obtained from each individual. 47 subjects covering five severity levels from healthy, gingivitis, slight, moderate-to-severe periodontitis sites were recruited from September to December 2021, in the Department of Periodontology, the Second Clinical Division, Peking University Hospital and School of Stomatology. All patients were systemically healthy and divided into five groups. The diagnosis of chronic periodontitis according to the 1999 classification of periodontal diseases and conditions [24]. The clinical inclusion criteria of different severity sites were as follows: severe periodontitis sites, PD ≥ 7 mm, CAL ≥ 5 mm, and BI ≥ 2; moderate periodontitis sites, PD = 4–5 mm, CAL = 3–4 mm, and BI ≥ 2; slight periodontitis sites, PD ≥ 4 mm, CAL = 1–2 mm, and BI ≥ 2; gingivitis sites, PD ≤ 3 mm, BI > 1. The healthy control group subjects were defined as those with good oral hygiene, calculus index = 0, no swelling of gums, PD ≤ 3 mm, BI ≤ 1.

The exclusion criteria were as follows: (1) use of anti-inflammatory drugs, antibiotics and immunosuppressants in the past six months; (2) systemic diseases that may interfere with the progression of periodontal diseases, such as diabetes and immune diseases; (3) gingival lesions unrelated to plaque; (4) use of orthodontic appliances; (5) pregnancy and lactation; (6) periodontal treatment in the past six months; (7) continuous use of mouthwash containing antibacterial agent in the past two months; (8) smoking; (9) the surface of attached gingiva and mucosa was stained.

### Spectra collection and preprocessing

The VNIR reflectance spectra were obtained with a handheld portable spectrometer HyScan1700-ZT (Progoo Information Technology Co., Ltd, Tianjin, China). In order for convenient chair side use in the oral cavity, three optical fibers with a diameter of 600 μm were coupled together at the probing end, where one fiber output highly stable light offered by a built-in 5-W tungsten halogen light source, and the other two fibers connecting with two spectrometers were used to collect reflected light from the tissue. Therefore, the HyScan1700-ZT can measure radiance in continuously 400–1700 nm wavelength range with a spectral resolution of about 3 nm @ 400–1000 nm and 10 nm @ 1000–1700 nm, using a smart phone connected with the spectrometer via Bluetooth and a special oral probe with 3.2 mm in diameter and 9.8 mm in length. The patients were seated in a relaxed, standard position on a dental chair during all spectral collections. The portable spectrometer probe was placed 3 mm away from the gingival tissue surface and spectral measurements were acquired at 6 sites of distal-buccal, buccal, mesial-buccal, mesial-lingual, lingual, distal-lingual, respectively. At the baseline level, all radiance spectra were collected prior to clinical measurements from 32 severe periodontitis sites, 31 moderate periodontitis sites, 31 slight periodontitis sites, 37 gingivitis sites and 55 healthy sites. All clinical parameters and spectra were obtained by the same examiners.

To eliminate the systematic measurement uncertainties, every 5 replicates were averaged in each site. A spectral standard panel with about 5% reflectance factor was simultaneously measured to calibrate the radiance into reflectance. Finally, in order to identify the spectral absorption features of interest as well as normalize all the spectral curves to a consistent measurement background, the continuum removal was conducted to all the spectral reflectance [25]. The continuum defined as the convex hull fit over the top of a spectrum using straight-line segments that connect local spectra maxima was derived using the ENVI image processing package (Exelis Visual Information Solutions, Inc., 2015), and then it was removed by dividing the original reflectance spectrum by the corresponding continuum curve across the whole wavelengths, as follows:

1 $$S_{cr} (\lambda ) = {\raise0.7ex\hbox{${S(\lambda )}$} \!\mathord{\left/ {\vphantom {{S(\lambda )} {C(\lambda )}}}\right.\kern-0pt} \!\lower0.7ex\hbox{${C(\lambda )}$}},$$

where $S_{cr} (\lambda )$ is the continuum-removal reflectance spectrum at the wavelength $\lambda$, $S(\lambda )$ and $C(\lambda )$, respectively, stand for the original reflectance spectrum and the continuum curve (convex hull) at the corresponding wavelength $\lambda$. The continuum-removal analysis was first proposed for mineral mapping, and afterwards it was widely used in various applications such as vegetation analysis and estimation of chemical concentrations due to its unique advantage in isolating spectral absorption signatures of interest from background absorptions [25].

### Periodontal examination

The clinical indicators including PD, CAL, BI and plaque index (PLI) were measured and recorded at 6 sites of distal-buccal, buccal, mesial-buccal, mesial-lingual, lingual, distal-lingual excluding the third molars, using a Williams periodontal probe (Hu-Friedy, Chicago, United States). Ten subjects in periodontitis group received corresponding supragingival scaling, subgingival scaling and root planing. The spectral measurements were acquired and the clinical examination were recorded from 52 periodontitis sites at baseline and 6 weeks after periodontal treatment. All spectral collection, clinical examinations and treatments were performed by one specially trained dentist from Peking University School of Stomatology, China, to assure both spectral and clinical data acquisitions consistent and reliable.

### Development of HSIs from continuum-removal reflectance

**Fig. 1:**
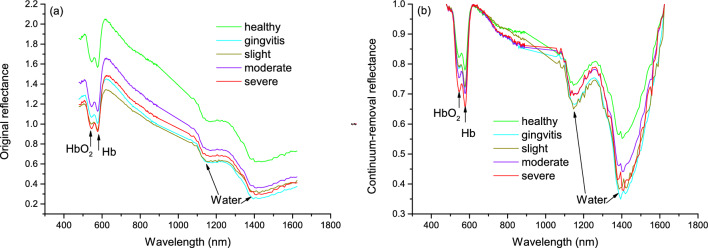
Spectra of periodontal tissues over five inflammatory conditions from healthy to severe periodontitis: **a** original reflectance spectra referenced to a 5% spectral on standard panel, **b** continuum-removal reflectance spectra

It is reported that HbO_2_ and Hb can cause obvious absorptions centered at about 544 nm and 576 nm, respectively, and the water in tissue can result in important absorptions in bands centered at about 1100 and 1450 nm [11, 26]. To facilitate the extracting of the above absorptions related to inflammation dynamics, continuum-removal analysis was introduced to quantify spectral absorption features, and the normalized spectral indices were constructed based on the continuum-removal reflectance, as follows:

2 $$D_{\lambda } = 1 - \rho_{\lambda } ,$$

3 $$ND{\text{HbO}}_{2} I = \frac{{D_{{{\text{HbO}}_{2} }} - D_{484} }}{{D_{{{\text{HbO}}_{2} }} + D_{484} }},$$

4 $$ND{\text{Hb}}I = \frac{{D_{{{\text{Hb}}}} - D_{617} }}{{D_{{{\text{Hb}}}} + D_{617} }},$$

5 $$t{\text{Hb}} = D_{{{\text{HbO}}_{2} }} + D_{{{\text{Hb}}}} ,$$

where $D_{\lambda }$ and $\rho_{\lambda }$ are, respectively, the depth and continuum-removal reflectance at the wavelength $\lambda$. Thereby, $D_{{{\text{HbO}}_{2} }}$,$D_{{{\text{Hb}}}}$ and $D_{{{\text{water}}}}$ were calculated at the maximum absorption wavelengths of HbO_2_ (~ 544 nm), Hb (~ 576 nm) and water (~ 1146 nm), respectively (Fig. 1b). Drawing on ideas of widely used normalized difference vegetation indices (NDVI) in remote sensing [27], two normalized difference hemodynamic spectral indices (called as $ND{\text{HbO}}_{2} I$ and $ND{\text{Hb}}I$) were developed, respectively, based on the left insensitive band (~ 484 nm) of HbO_2_ and the right insensitive band (~ 617 nm) of Hb for better indicating dynamics of oxyhemoglobin and deoxyhemoglobin of periodontal tissues. The tHb was calculated as the sum of $D_{{\text{HbO}_{2} }}$ and $D_{\text{Hb}}$, used as a proxy of the total hemoglobin of gingival tissue.

### Statistical analyses

All the HSIs were analyzed, respectively, over five severity levels (healthy, gingivitis, slight periodontitis, moderate periodontitis and severe periodontitis) using a one-way analysis of variance (ANOVA). Additionally, the HSIs of before and after therapy groups of sites were analyzed by t-test. Finally, the correlations between periodontal clinical parameters (PD, CAL, BL and PLI) and the proposed HSIs were performed by Spearman correlation coefficients. Data processing and analysis was performed with SPSS 24.0 (IBM, Armonk, NY, USA). Differences were considered significant at the 0.05 level (i.e., *P* < 0.05).

## Results

### Statistical results for clinical parameters from groups under different severities

The mean age of the recruited subjects was 34.5 ± 7.2 years, with a range from 20 to 58 years old, and 18 (38.3%) of the subjects were male, 29 (61.7%) were female. As shown in Table 1, the clinical parameters (PD, CAL, BI and PLI) were presented as the mean ± standard deviation. Regarding PD, BI and PLI, the means of gingivitis sites, slight periodontitis, moderate periodontitis and severe periodontitis were significantly different from the healthy group (*P* < 0.05), whereas CAL exhibited no statistically significant between healthy and gingivitis. However, all the clinical parameters after periodontal therapy differed from that before therapy (*P* < 0.05).

**Table 1:** Statistical analyses over clinical parameters from each group

Groups	PD (mm)	CAL (mm)	BI	PLI
Healthy (*n* = 9)	1.80 ± 0.16	0.20 ± 0.08	0.66 ± 0.24	0.51 ± 0.37
Gingivitis (*n* = 9)	3.06 ± 0.29*	0.33 ± 0.16	2.02 ± 0.05*	1.03 ± 0.35*
Slight periodontitis (*n* = 11)	4.00 ± 0.00*	1.48 ± 0.41*	1.81 ± 0.63*	1.43 ± 0.45*
Moderate periodontitis (*n* = 10)	5.30 ± 0.36*	2.89 ± 1.08*	2.06 ± 0.18*	1.28 ± 0.43*
Severe periodontitis (*n* = 8)	7.32 ± 0.47*	7.03 ± 0.93*	2.68 ± 0.78*	1.36 ± 0.39*
Before therapy (*n* = 10)	4.46 ± 0.48	2.59 ± 1.65	1.94 ± 0.53	1.27 ± 0.36
After therapy (*n* = 10)	2.84 ± 0.15*	0.51 ± 0.85*	1.22 ± 0.23*	0.90 ± 0.26*

PD: probing depth; CAL: clinical attachment loss; BI: bleeding index on probing; PLI: plaque index. Statistical measures of variations for clinical parameters were presented by mean ± standard deviation. Statistical significance at the 0.05 level denoted by *

### Spectral properties for periodontal tissues over five inflammatory conditions

The radiance spectra of periodontal tissues over different inflammatory conditions were calibrated to reflectance by a spectral standard panel with a reflectance factor of 5% across the 400–1700 nm (Fig. 1a). It was shown that two strong absorption features caused by hemoglobin within the 500–600 nm spectral region as well as the two typical primary water absorptions at about 1100 and 1450 nm were visually distinguishable among all the inflammatory conditions from healthy to severe periodontitis. Specifically, according to previous studies, the absorptions of light by HbO_2_ dominated shorter wavelengths centered at about 544 nm and Hb induced longer absorptions centered at about 576 nm. Both together caused the obvious and unique absorption peeks that are closely related to hemoglobin dynamics of periodontal tissues in the visible spectral range. However, the change trend of original reflectance spectra showed much confused across the five inflammation levels, probably due to deviated measurement backgrounds spanning four months. As shown in Fig. 1b, after removing the continuum of the original spectrum, the continuum-removal reflectance spectra were well normalized to a consistent range within 0 to 1, so that we can compare individual absorption features from a common baseline. Compared with the original reflectance spectra in Fig. 1a, the absorption features of interest were greatly highlighted in the continuum removed spectra of Fig. 1b, and the absorption depths generally increased as inflammation progressed from healthy to severe periodontitis, particularly in the vicinity of the two hemoglobin-dominated absorption bands (i.e., 544 nm and 576 nm).

### Performances of periodontal HSIs

**Fig. 2:**
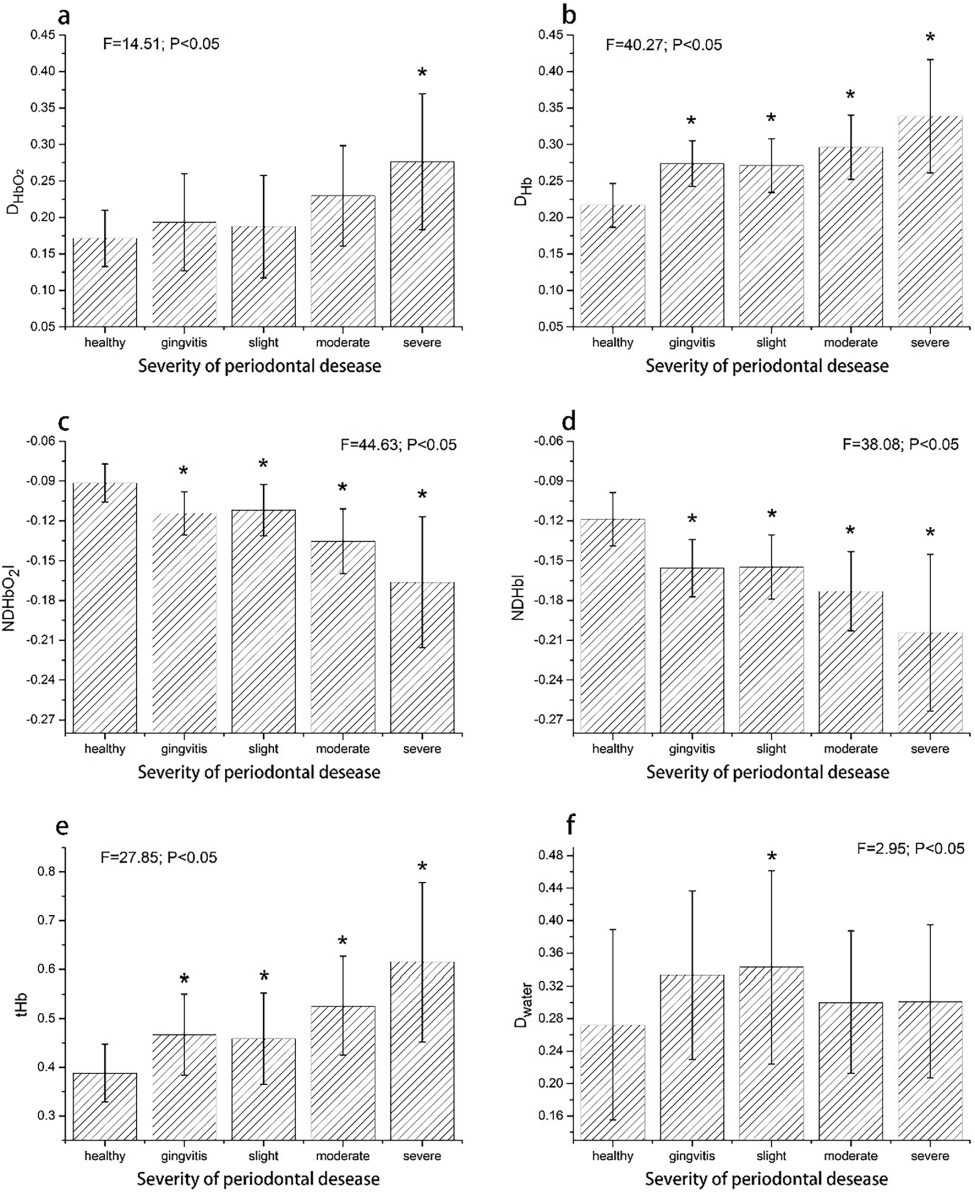
Mean comparisons of $D_{{{\text{HbO}}_{2} }}$(**a**), $D_{{{\text{Hb}}}}$(**b**), $ND{\text{HbO}}_{2} I$ (**c**), $ND{\text{Hb}}I$ (**d**), $t{\text{Hb}}$(**e**), $D_{{{\text{water}}}}$ (**f**) for every periodontal disease severity level. Analysis of variance of each spectral index was conducted. $ND{\text{HbO}}_{2} I$, normalized difference index of oxyhemoglobin; $ND{\text{Hb}}I$, normalized difference index of deoxyhemoglobin; $t{\text{Hb}}$, $D_{{{\text{Hb}}}}$, $D_{{{\text{HbO}}_{2} }}$, $D_{{{\text{water}}}}$ represent spectral absorption depths of total hemoglobin, deoxyhemoglobin, oxyhemoglobin and tissue water, respectively. “*” indicates significant differences from the healthy group at *P* < 0.05. Error bars indicate standard deviation

Six HSIs including $D_{{{\text{HbO}}_{2} }}$, $D_{{{\text{Hb}}}}$, $ND{\text{HbO}}_{2} I$, $ND{\text{Hb}}I$, $t{\text{Hb}}$, $D_{{{\text{water}}}}$ were separately calculated as absorption indicators using the continuum removed reflectance spectra. Figure 2 presents mean comparisons of the six HSIs for every periodontal disease severity level. $D_{{{\text{HbO}}_{2} }}$ exhibited a slight increase trend as disease severity increased, showing significant differences (*P* < 0.05) from the healthy sites at moderate and severe groups (Fig. 2a). $D_{{{\text{Hb}}}}$ and $t{\text{Hb}}$ were lowest (*P* < 0.05) in healthy groups, and increased steadily with the increase in inflammation severity (Fig. 2b and e). By contrast, $NDHbO_{2} I$ and $NDHbI$ showed significantly downward trends (*P* < 0.05) as severity increased (Fig. 2c, d). However, no significant differences in water absorption depth existed due to periodontal inflammation (Fig. 2f). These results showed that $D_{{{\text{Hb}}}}$, $t{\text{Hb}}$, $ND{\text{HbO}}_{2} I$ and $ND{\text{Hb}}I$ were sensitive to the inflammation severity, and had great potentials for early detection of periodontitis, while $D_{{HbO_{2} }}$ was a good indicator to probe the presence of moderate-to-severe periodontitis. The relative tissue water content was unable to indicate the periodontal inflammation development, which was in agreement with other studies [9], although it increased from healthy stage to slight periodontitis (*P* < 0.05).

**Fig. 3:**
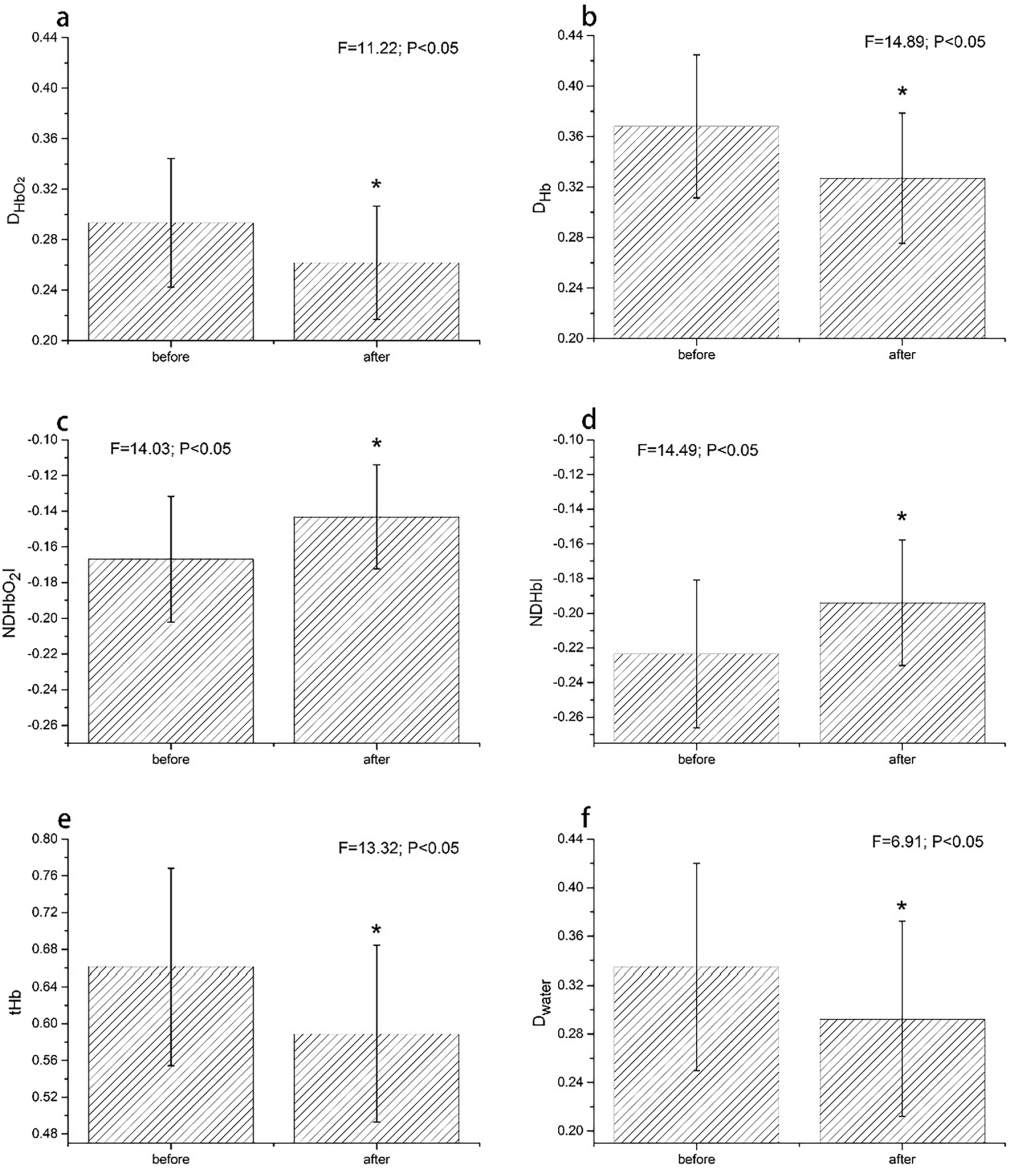
Mean comparisons of $D_{{{\text{HbO}}_{2} }}$ (**a**), $D_{{{\text{Hb}}}}$ (**b**), $ND{\text{HbO}}_{2} I$ (**c**), $ND{\text{Hb}}I$ (**d**), $t{\text{Hb}}$ (**e**), $D_{{{\text{water}}}}$ (**f)** for before and after periodontal therapy. Analysis of variance of each spectral index was conducted. $NDHbO_{2} I$: normalized difference index of oxyhemoglobin; $ND{\text{Hb}}I$: normalized difference index of deoxyhemoglobin; $t{\text{Hb}}$, $D_{{{\text{Hb}}}}$, $D_{{{\text{HbO}}_{2} }}$, $D_{{{\text{water}}}}$ represent spectral absorption depths of total hemoglobin, deoxyhemoglobin, oxyhemoglobin and tissue water, respectively. “*” indicates significant differences from the healthy group at *P* < 0.05. Error bars indicate standard deviation

The performances of the HSIs were also confirmed in evaluating the effect of periodontitis therapy before and after treatment. As shown in Fig. 3, all six gingival tissue spectral indices (HSIs) showed significant differences (*P* < 0.05).

### Relationship between periodontal HSIs and clinical parameters

The Spearman correlation coefficients between HSIs ($D_{{{\text{HbO}}_{2} }}$,$D_{{{\text{Hb}}}}$, $t{\text{Hb}}$, $ND{\text{HbO}}_{2} I$ and $ND{\text{Hb}}I$) and the clinical parameters (PD, CAL, BI and PLI) are listed in Table 2. Both $ND\text{HbO}_{2} I$ and $ND\text{Hb}I$ exhibited significantly negative correlations with all the clinical parameters (*P* < 0.05), whereas the other three HSIs ($D_{{{\text{HbO}}_{2} }}$, $D_{{{\text{Hb}}}}$ and $t{\text{Hb}}$) showed positive correlations at the 0.05 significance level. Among the five HSIs, $ND\text{HbO}_{2} I$ and $ND\text{Hb}I$ yielded the highest correlations with PD (*r* = − 0.491 and *r* = − 0.444), CAL (*r* = − 0.591 and *r* = − 0.529), BI (*r* = − 0.315 and *r* = − 0.296) and PLI (*r* = − 0.203 and *r* = − 0.215), respectively. Additionally, the HSIs were more correlated with PD and CAL than BI and PLI.

**Table 2:** Correlations between HSIs and clinical parameters

HSIs	PD (mm)	CAL (mm)	BI	PLI
${{\text{NDHbO}}}_{2}{\text{I}}$	− 0.491*	− 0.591*	− 0.315*	− 0.203*
${\text{NDHbI}}$	− 0.444*	− 0.529*	− 0.296*	− 0.215*
${\text{tHb}}$	0.408*	0.510*	0.258*	0.195*
${{\text{D}}}_{{\text{Hb}}}$	0.441*	0.525*	0.297*	0.210*
${{\text{D}}}_{{{\text{HbO}}}_{2}}$	0.420*	0.529*	0.261*	0.190*

HSIs, hemodynamic spectral indices; NDHbO2I, normalized difference index of oxyhemoglobin; NDHbI, normalized difference index of deoxyhemoglobin; tHb,${{\text{D}}}_{{\text{Hb}}}$, ${{\text{D}}}_{{{\text{HbO}}}_{2}}$ represent spectral absorption depths of total hemoglobin, deoxyhemoglobin and oxyhemoglobin, respectively; PD: probing depth; CAL: clinical attachment loss; BI: bleeding index on probing; PLI: plaque index. Statistical significance at the 0.05 level denoted by *

## Discussion

The conventional diagnostic techniques (clinical and radiographic parameters) have always been routinely used for periodontal disease diagnosis, which helped define the extent and severity of periodontitis. Clinical parameters such as BOP, PD and CAL were not sensitive due to many confounding factors. For instance, the intensity and direction of periodontal probing, the inflammatory status of the tissue and the poor reliability and reproducibility of measuring CAL may affect its diagnostic accuracy [28, 29]. In addition, the traditional method needs professional operation and is time-consuming, which is not suitable for large-scale epidemic investigation. In addition, radiographs only show changes in the bones when as much as 30% to 50% of the mineral has already been lost [30, 31]. Most importantly, clinical probing and radiographs mainly reflected a historical perspective of the disease status. Therefore, some new inflammatory parameters such as pocket oxygen tension, temperature and oxygen saturation of hemoglobin, have been proposed as potential periodontal diagnostic indicators for evaluating the status of periodontal tissue [3, 32, 33]. However, these new methodologies are often inconvenient or impractical for routine chairside usage.

For these reasons above, more accurate and efficient methods are urgently needed for periodontal clinical diagnostics of patients by offering earlier, precise, less-invasive and cost-effective therapies [10, 11, 34]. VNIR spectroscopy has been proposed to have the potential to non-destructively assess periodontal inflammation [9]. In our study, we provided increased insights into spectral behaviors across the 400–1700 nm wavelength range for periodontal tissues over five inflammation stages, and on this basis, we developed six HSIs ($D_{{{\text{HbO}}_{2} }}$, $ND{\text{HbO}}_{2} I$, $D_{{{\text{Hb}}}}$, $ND{\text{Hb}}I$, $t{\text{Hb}}$ and $D_{{{\text{water}}}}$) from continuum-removal reflectance spectra to explore their potential for early and precision detection for periodontal inflammation. To do this, to avoid possible interferences, patients presenting with confounding risk factors for periodontal diseases were excluded, and then all the subjects were divided into five groups from healthy, gingivitis, slight, moderate to severe periodontitis according to the periodontal disease severity. We found that continuum-removal analysis effectively suppressed the original spectral noises, reduced measurement uncertainties, and highlighted spectral absorption features, which was conducive to comparisons analyses under different spectral measurement backgrounds and times. Our results showed that $D_{{{\text{HbO}}_{2} }}$, $D_{{{\text{Hb}}}}$ and $t{\text{Hb}}$ presented gradual increase trends from healthy sites to severe periodontitis. This may be mainly attributed to that both the oxygen supply and the oxygen consumption increased during the inflammation development [35]. However, the increase of the relative deoxyhemoglobin was greater than that of oxyhemoglobin, consistent with previous studies [11, 36]. It’s reported that proliferative and reparative changes during inflammation may cause tissue hypoxia due to the increased oxygen consumption. In addition, vascular damage, large numbers of infiltrating cells and intensive metabolic activity of pathogens also can result in hypoxia in gingiva [11, 34]. Total hemoglobin, closely related to the tissue blood volume, may be a further prognostic indicator of periodontal disease, because periodontitis is an inflammation closely associated with vasodilation and angiogenesis [9, 21].

Compared to the absorption depth indicators ($D_{{{\text{HbO}}_{2} }}$, $D_{{{\text{Hb}}}}$ and $t{\text{Hb}}$), the normalized difference spectral indices ($ND{\text{HbO}}_{2} I$ and $ND{\text{Hb}}I$) conveyed more steadily decreasing trend (*P* < 0.05) as the inflammation increased from healthy to periodontitis sites (Fig. 2c, d). These HSIs were also further evaluated using an independent group of clinical data, which were proved to be useful for assessing the effectiveness of periodontal treatment (Fig. 3). We also found that $D_{{{\text{water}}}}$ (a proxy of tissue water content) increased from healthy to slight periodontitis, but decreased at moderate and severe periodontitis. That may be because the main clinical signs of gingivitis and slight periodontitis are swelling and redness due to local edema and inflammation in active inflammatory process. However, when the disease proceeded to moderate and severe periodontitis, the primary pathological changes were characterized by destruction of the supporting bone along with a certain degree of inflammatory infiltrate. Nevertheless, the $D_{{{\text{water}}}}$ showed unstable sensitives to inflammation severity due to spectral uncertainties caused by water strong absorptions of light.

Finally, correlations between the periodontal HSIs and clinical indicators were separately conducted. This study proved the potentials of all the HSIs for indicating periodontal inflammatory, and the two normalized difference spectral indices ($ND{\text{HbO}}_{2} I$ and $ND{\text{Hb}}I$) exhibited higher correlations than the spectral absorption depth indicators ($D_{{{\text{HbO}}_{2} }}$, $D_{{{\text{Hb}}}}$ and $t{\text{Hb}}$) with PD, CAL, BI and PLI. Compared to that obtained by previous studies [9, 21], we greatly improved the correlations between HSIs and clinical parameters by constructing normalized difference hemodynamic spectral indices based on continuum-removal reflectance. For example, the $NDHbO_{2} I$-PD correlation was about − 0.49, much higher than the oxyhemoglobin-PD correlation (about − 0.18) in earlier research [9].

## Conclusions

In conclusion, we demonstrated great potentials of VNIR spectroscopy for detection and precision diagnosis of periodontal diseases through extracting robust spectral absorption features that were considered to be closely related to the dynamics of deoxyhemoglobin and oxyhemoglobin at five stages of inflammation development from healthy, gingivitis, slight, moderate-to-severe periodontitis. This study will pave the way toward improving understanding of linkage between spectral behaviors and periodontal pathogen infection during the inflammation development, and it will be particularly beneficial to develop optimal models and smart devices to painlessly, non-invasively and instantly probe periodontitis at earlier stages.

## Data Availability

Data are available upon reasonable request.

## Abbreviations

BI: Bleeding index
BOP: Bleeding on probing
CAL: Clinical attachment loss
D_HbO2_: Spectral absorption depths of oxyhemoglobin
D_Hb_: Spectral absorption depths of deoxyhemoglobin
HbO_2_: Oxyhemoglobin
Hb: Deoxyhemoglobin
NDHbO_2_I: Normalized difference indices of oxyhemoglobin
NDHbI: Normalized difference indices of deoxyhemoglobin
PD: Probing depth
PLI: Plaque index
tHb: Total hemoglobin
HSIs: Hemodynamic spectral indices
VNIR: Visible and near-infrared spectroscopy
